# Cold Atmospheric Plasma Disrupts Microbial Wound Bioburden: In Vitro, Porcine MRSA Biofilm, and Clinical Fluorescence Evidence from Bench to Bedside

**DOI:** 10.3390/biomedicines14081750

**Published:** 2026-08-03

**Authors:** Steven Jeffery, Ahmad Wakaf, Lennart Marlinghaus, Sören Gatermann, Thomas Meyer, Joel Gil, Ivan Jozic, Stephen C. Davis

**Affiliations:** 1 Pioneer Wound Healing and Lymphoedema Centres (Pioneer-WHLC), Eastbourne BN23 8AS, UK; steven.jeffery@pioneer-whlc.org; 2 Wound Healing Practice Development Unit, Birmingham City University, Birmingham B5 5JU, UK; 3 Division of Surgery and Interventional Science, University College London (UCL), London WC1E 6BT, UK; ahmad.wakaf.23@ucl.ac.uk; 4 Department of Medical Microbiology, Institute for Hygiene and Microbiology, Ruhr-University Bochum, Universitätsstraße 150, 44892 Bochum, Germany; lennart.marlinghaus@rub.de (L.M.); soeren.gatermann@rub.de (S.G.); 5 Klinik für Dermatologie, Venerologie und Allergologie, Ruhr-Universität Bochum, St. Josef Hospital, Gudrunstr. 56, 44791 Bochum, Germany; thomas.meyer@klinikum-bochum.de; 6 Wound Healing Research Program, Dr. Phillip Frost Department of Dermatology and Cutaneous Surgery, University of Miami Miller School of Medicine, Miami, FL 33136, USA; jgil@med.miami.edu (J.G.); i.jozic@med.miami.edu (I.J.)

**Keywords:** cold atmospheric plasma (CAP), chronic wounds, biofilm, MRSA, *Candida albicans*, fluorescence imaging, wound healing, reactive oxygen species

## Abstract

**Background:** Biofilm-associated microbial burden is a major barrier to wound healing and is increasingly difficult to control in the era of antimicrobial resistance. This translational study evaluated the antimicrobial performance and tissue compatibility of a commercially available large-area cold atmospheric plasma (CAP) system (CPTpatch^®^/CPTcube^®^; Coldplasmatech GmbH, Greifswald, Germany) across in vitro, porcine, and clinical patient settings. **Methods:** CAP was tested against seven wound-relevant bacterial species, including multidrug-resistant strains, and the fungus *Candida albicans* in vitro, in two porcine MRSA-infected deep dermal wound models, and in a clinical service-evaluation cohort of 20 chronic lower-limb wounds in 14 patients; 17 wounds had evaluable longitudinal MolecuLight^®^ (Toronto, ON, Canada) fluorescence series. **Results:** In vitro, CAP induced rapid multi-log killing across the bacterial panel and showed a marked antifungal effect against *C. albicans*. In porcine MRSA wounds, CAP reduced bacterial burden versus sham by 1.65, 1.89, and 2.04 log10 CFU/g on Days 4, 8, and 11, equivalent to 97.8%, 98.7%, and 99.1% reductions. In a 72-h post-inoculation MRSA biofilm model, the strongest 5×/week regimen achieved 2.51 log10 (99.69%) and 3.18 log10 (99.93%) reductions versus baseline after 2- and 4-min CAP exposures, respectively. Clinically, twice-weekly CAP was associated with a significant decline in MolecuLight^®^ fluorescence grades over 5 weeks (*p* < 0.001); effective surface disinfection at 5 weeks was observed in 16/17 evaluable wounds. **Conclusions:** From bench to bedside, the investigated CAP system delivered rapid broad-spectrum antimicrobial activity, reduced MRSA burden in biofilm-infected porcine wounds, and was associated with clinically measurable suppression of wound surface bioburden while remaining tissue-sparing. These findings support further controlled studies to define clinical benefit in wound care.

## 1. Introduction

Acute and chronic wounds constitute a significant and increasing global healthcare burden. Chronic wounds alone are estimated to affect approximately 1–2% of the population in developed countries [1]. In the United States, more than 6 million people are affected by acute and chronic wounds with an annual cost of over $25 billion [2]. Further, the global healthcare burden was estimated at $148.65 billion in 2022 [3].

Unlike acute wounds, which typically follow a predictable healing trajectory, chronic wounds are characterized by delayed or stalled healing due to a complex interplay of factors. These include persistent inflammation, ischemia, metabolic dysregulation and, critically, microbial colonization and biofilm formation. Biofilms create a protective microenvironment that shields pathogens from host immune responses and antimicrobial agents, contributing to treatment resistance and chronicity [4,5]. Despite the availability of advanced wound care modalities, biofilm-associated infections remain a major barrier to healing. Moreover, many conventional antiseptics and antibiotics exhibit cytotoxicity toward host cells, further complicating tissue repair. The escalating threat of antimicrobial resistance underscores the urgent need for novel, non-antibiotic, and non-cytotoxic strategies to manage wound bioburden effectively [6,7,8,9,10,11,12].

Biofilms also contain metabolically heterogeneous bacterial populations, including slow-growing and persister-like cells, which can tolerate antimicrobial exposure even in the absence of classical genetic resistance [13,14]. This biofilm-associated tolerance sustains local inflammation, increases bacterial and host-derived proteolytic burden, impairs keratinocyte and fibroblast function, and delays granulation and re-epithelialization [11,15,16,17,18]. These features are clinically relevant in chronic wounds, where microbial bioburden, biofilm-associated tolerance, and antimicrobial resistance may coexist and contribute to delayed healing and treatment failure [6,10,12].

Current management of wound bioburden relies on wound bed preparation, including repeated cleansing and debridement, topical antiseptics, antimicrobial dressings, and systemic antibiotics when clinical infection is present [19,20,21]. However, these strategies may be limited by biofilm-associated tolerance, antimicrobial resistance, local cytotoxicity, or incomplete access to microorganisms embedded within the wound microenvironment [4,5,6,7,8,9]. Cold atmospheric plasma (CAP) provides a complementary non-antibiotic approach, but clinical CAP should not be regarded as a uniform technology. Plasma jets, plasma torches, and DBD-based systems differ in plasma generation, feed gas, treatment distance, applicator geometry, treated surface area, and exposure standardization [22]. Selected point-source and small-area systems in the peer-reviewed literature report effective treatment fields from approximately 1 cm^2^ for plasma-jet applications to approximately 27.5 cm^2^ for DBD electrodes [23,24]. By contrast, the CPT platform evaluated here provides a sealed patch-based DBD treatment field of 11 × 11 cm, corresponding to 121 cm^2^, with a pre-programmed 2-min cycle and fixed operating parameters. The CPT patch creates an occlusive ambient-air compartment between the plasma-generating foil and the wound surface. Ionization within this confined volume helps limit dispersion of plasma-generated reactive species and supports a controlled local exposure profile. Compared with non-occlusive or open-air CAP systems, which may be more affected by treatment distance, airflow, applicator movement, and operator technique, the sealed patch-based CPT configuration may provide a more reproducible and less operator-dependent exposure over the defined treatment field. Large-area CPT-based delivery has also been adapted to treatment-bag configurations for extensive burn surfaces, including near-whole-body exposure over three consecutive 2-min cycles in a multimodal burn-care case report [25]. Because bacterial reduction varies between CAP sources, device designs, treatment areas, and exposure protocols, results from heterogeneous plasma systems should not be generalized across all CAP modalities; a systematic review and meta-analysis reported an acceptable safety profile but no significant pooled bioburden reduction across earlier atmospheric plasma studies [26]. The present study therefore evaluates a standardized large-area CPT DBD platform across in vitro, porcine MRSA biofilm, and clinical fluorescence settings.

Cold atmospheric plasma (CAP) has emerged as a promising adjunct in wound management, particularly for antimicrobial applications. CAP generates a mixture of reactive oxygen and nitrogen species (RONS), UV photons, charged particles, and electric fields that disrupt bacterial membranes, damage microbial DNA, and degrade substances that stabilize biofilms. Importantly, CAP operates at non-thermal temperatures and can be applied directly at the wound surface, minimizing host tissue damage [27,28,29]. Early laboratory studies have demonstrated rapid bactericidal activity against a broad range of organisms, including antibiotic-resistant strains such as methicillin-resistant *Staphylococcus aureus* (MRSA) [30,31]. Early clinical data, including two randomized controlled trials using argon-based CAP devices, reported significant reductions in bacterial load in chronic wounds with excellent safety [32,33].

Beyond antimicrobial action, RONS also participate in signaling pathways relevant to wound healing, angiogenesis, and re-epithelialization [34,35,36,37,38]. Preclinical and early clinical work with CAP has suggested that CAP may also modulate inflammation and early tissue repair, features particularly important in wound management [37,38,39,40]. However, despite encouraging mechanistic and preclinical data, there remains a lack of integrated evidence linking in vitro antimicrobial efficacy, in vivo biofilm clearance, and clinically meaningful outcomes in human wounds.

Therefore, the objective of this translational study was to evaluate whether a standardized large-area CPTpatch^®^/CPTcube^®^ cold atmospheric plasma system can reduce microbial wound bioburden across complementary in vitro, porcine MRSA biofilm, and clinical patient settings, while preserving tissue compatibility. We hypothesized that CAP would produce reproducible antimicrobial effects against clinically relevant bacterial and fungal pathogens, including resistant organisms, and would reduce bacterial fluorescence in chronic wounds without treatment-related tissue damage.

## 2. Materials and Methods

### 2.1. Cold Atmospheric Plasma Device and Operating Parameters

All experiments utilized the CPTpatch^®^/CPTcube^®^ dielectric barrier discharge (DBD) large area cold atmospheric plasma system (Coldplasmatech GmbH, Greifswald, Germany). The CPT ColdPlasma system is used as an adjunct therapy in the treatment and management of wounds by generating a cold, atmospheric pressure plasma in a sealed space or volume filled with ambient air around the wound surface.

Specifically, cold atmospheric plasma was generated using a dielectric barrier discharge (DBD) plasma foil applicator (CPTpatch^®^) connected to a control unit (CPTcube^®^). The plasma foil was applied occlusively over the target area using an adhesive frame, creating a closed air volume above the wound surface. The nominal treatment area defined by the adhesive frame was 11 × 11 cm, and the electrode layout was designed to provide a plasma footprint corresponding to this area. The control unit delivered a kV-range excitation signal to the patch (7 kV peak to peak/3.5 kV amplitude) at 6–7 kHz, with pulse modulation at 5 Hz, resulting in an average electrical power coupled into the plasma of approximately 10 W. Treatments were performed in ambient room air at room temperature without active control of environmental humidity. With direct application, the patch-to-wound spacing was typically 0.5 cm for a flat wound surface. According to conformity testing documentation, maximum patch temperature under standard operation remained <41 °C.

A 2-min standard treatment cycle is pre-programmed, ensuring consistent plasma dosing across studies. Operating parameters including the voltage waveform, pulse frequency, plasma composition, and thermal profile were kept identical across the in vitro, porcine, and clinical components of this study, ensuring translational alignment. Only treatment duration was modified to allow adequate exposure time.

### 2.2. In Vitro Antimicrobial Testing

Eight clinically relevant wound pathogens were selected for evaluation, encompassing both Gram-negative and Gram-positive bacteria, as well as a fungal species. The Gram-negative organisms included *Escherichia coli* (NRZ-14408), *Klebsiella pneumoniae* (NRZ-24615), *Acinetobacter baumannii* (NRZ-24584), *Pseudomonas aeruginosa* (NRZ-24555) while the Gram-positive group comprised *Staphylococcus aureus* (ANR783381), *Staphylococcus lugdunensis* (DSM 4804) and *Enterococcus faecium* (ANR832735). In addition, the yeast *Candida albicans* (DSM 11948) was included to assess antifungal efficacy. Several of these strains carried clinically important resistance determinants, for example blaKPC, blaOXA, vanA, and mecA, reflecting organisms commonly found in chronic wound environments.

Strains from cryopreserved stocks were streaked onto cystine–lactose–electrolyte-deficient (C.L.E.D.) agar containing 1.5% agar and incubated for 24 h at 37 °C. Bacterial cells were collected from the plates using sterile inoculation loops and suspended in 0.9% NaCl solution. The optical density at 600 nm (OD600) was adjusted to the desired value, and 10-fold serial dilutions were prepared for CFU determination and plasma treatment.

Aliquots of 100 µL from each suspension were plated on C.L.E.D. agar plates using a Drigalski spatula. To determine the relationship between OD600 and CFU counts, serial dilutions of the inoculum ranging from approximately ($5\times 10^{8}$) to ($5\times 10^{2}$) CFU/mL were plated, as the OD–CFU relationship may vary between bacterial species. These plates also served as untreated controls.

The CPTpatch dielectric barrier discharge (DBD) cold atmospheric plasma device was used to expose the inoculated plates under standardized laboratory conditions. The inoculated agar plates were exposed to cold plasma for 10, 60, or 180 s and subsequently incubated for 24 h at 37 °C. CFU reduction was determined by comparing colony counts on untreated control plates with those on plasma-treated plates.

ATCC strains were not used exclusively because the aim of this study was to evaluate antimicrobial activity against clinically relevant wound pathogens, including organisms with resistance determinants commonly encountered in chronic wound infection. Therefore, the panel included repository and clinical/reference isolates with documented identifiers and resistance markers.

### 2.3. Porcine MRSA Biofilm Wound Model

A porcine model was used due to the morphological similarities between swine skin and human skin [41,42,43]. Two young female specific-pathogen-free Yorkshire pigs (2–3 months old, weighing 40–45 kg) were used in this study and were housed in AAALAC-accredited facilities at the University of Miami. Animals were allowed to acclimate to the housing environment for at least 5 days prior to the start of experimental procedures and were monitored daily for any observable signs of pain or distress throughout the study period. Analgesics were administered as appropriate to minimize discomfort during the entire experiment, in accordance with institutional animal care guidelines. All procedures were approved by the University of Miami Institutional Animal Care and Use Committee (IACUC) and conducted in accordance with institutional and federal guidelines.

**Figure 1:**
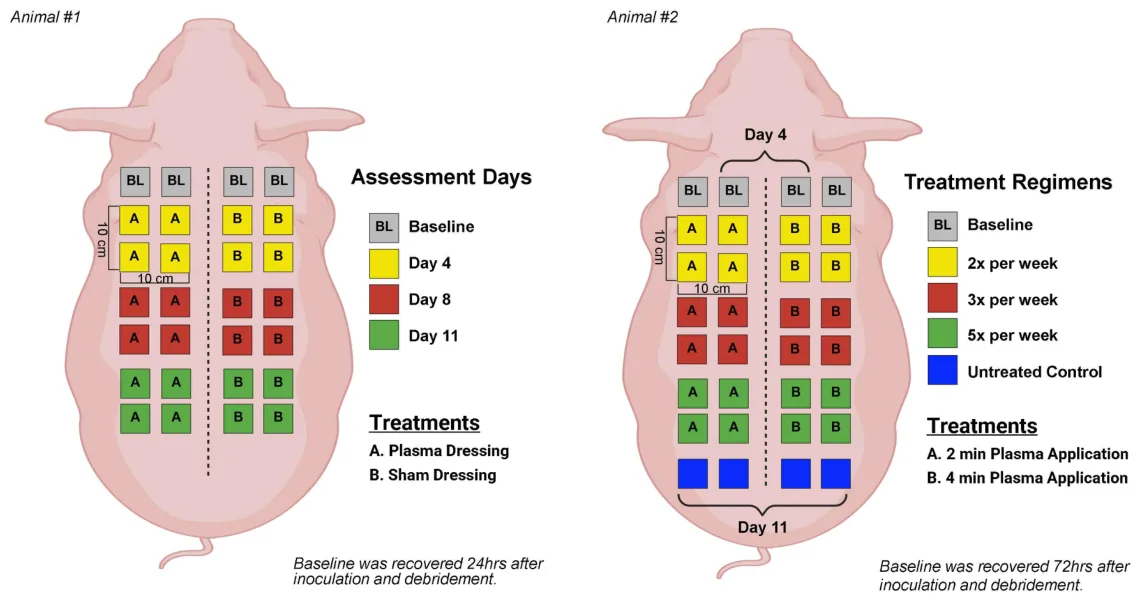
Experimental design of the porcine MRSA biofilm wound studies. In Animal #1, 28 wounds were created: 12 wounds were randomized to active CAP treatment, 12 wounds to sham dressing, and 4 wounds were reserved for baseline microbial quantification. In Animal #2, 32 wounds were allocated across CAP duration/frequency regimens, untreated controls, and baseline biopsies.

Deep dermal wounds (22 mm × 22 mm × 3 mm) were created on the paravertebral and thoracic regions using an electrokeratome. In Animal #1, 28 wounds were generated and randomly assigned to two treatment groups (n = 12 per group), with four wounds reserved for baseline microbial quantification. In Animal #2, 32 wounds were created and randomly assigned across six CAP treatment regimens defined by treatment duration (2 or 4 min) and frequency (2×, 3×, or 5× per week) (n = 4 wounds per regimen), with four wounds serving as untreated controls and four assigned for baseline sampling (Figure 1).

All wounds were inoculated with a standardized suspension of methicillin-resistant *Staphylococcus aureus* (MRSA USA-300) to establish biofilm. Baseline bacterial burden was assessed by recovering four wounds per animal at 24 h (Animal #1) or 72 h (Animal #2) post-inoculation. In Animal #1, wounds were treated with either an active plasma dressing (CPTpatch) or a sham dressing (non-adherent gauze) on Days 0, 4, and 8. In Animal #2, wounds received active plasma treatment at varying frequencies (2×, 3×, or 5× per week) and durations (2 or 4 min). For 4-min treatments, the CPTpatch device was operated for two consecutive 2-min cycles. Following each treatment, the plasma dressing was removed, and all wounds were covered with a non-adherent gauze dressing secured with surgical tape. Wound appearance was documented photographically, and erythema was scored daily using a predefined 5-point ordinal scale 1–5: 1 = absent, 2 = mild, 3 = moderate, 4 = marked, and 5 = exuberant.

On Day 0, four wounds per animal were biopsied (6 mm punch) post-debridement to establish baseline microbial counts. In Animal #1, four wounds per treatment group were biopsied on Days 4, 8, and 11. In Animal #2, two wounds per treatment regimen were biopsied on Days 4 and 11. Tissue samples were homogenized, plated, and incubated aerobically at 37 °C for 24–48 h. Viable colonies were enumerated, and bacterial load was expressed as CFU/g of tissue. Samples were fixed in formalin and subsequently stained with hematoxylin and eosin (H&E), Masson’s Trichrome, or Toluidine Blue. Additional incisional biopsies were collected from each wound on Days 4, 8, and 11 (Animal #1) and Days 4 and 11 (Animal #2) for histological assessment. All specimens were evaluated in a blinded manner using light microscopy to assess key histological features indicative of treatment response. Evaluated parameters included percent re-epithelialization, epithelial thickness, inflammatory cell infiltration, angiogenesis, granulation tissue formation, necrosis, fibrosis, and fatty infiltration. Histological scoring criteria are detailed in Supplementary Tables S1 and S2.

### 2.4. Clinical Evaluation in Chronic Wounds

A prospective observational clinical evaluation was conducted in 14 patients with 20 chronic lower-limb wounds present for more than six weeks despite standard wound care. Wound types comprised 15 venous ulcers, 2 mixed lymphovenous ulcers, 1 arterial ulcer, 1 lymphatic ulcer, and 1 traumatic wound. Patients were recruited from outpatient wound clinics and managed according to routine clinical practice, with CAP therapy added as an adjunctive intervention. Detailed baseline patient and wound characteristics, wound-level dataset completeness, and concomitant standard of care are provided in the Supplementary Materials Tables S3–S8. This study was conducted as a service evaluation of adjunctive CAP use in routine wound-care practice and was not designed as a population-representative clinical trial.

CAP therapy was delivered using the CPT system and applied directly to the wound surface. Treatments were administered twice weekly over a five-week intervention period for a period of two minutes. Treatment technique, exposure time, and device positioning were standardized across all sessions. Wounds were debrided at each dressing change prior to CAP application, and venous ulcers were managed with compression therapy.

Bacterial burden was assessed using the MolecuLight^®^ (Toronto, ON, Canada), a handheld fluorescence imaging device. Imaging was performed immediately prior to CAP application. Red fluorescence was interpreted as indicative of elevated bacterial burden exceeding approximately $10^{4}$ CFU per gram of tissue [44]. A standardized semi-quantitative fluorescence grading system was applied to all interpretable images. Fluorescence was classified as Grade 0 (no fluorescence), Grade 1 (localized fluorescence), or Grade 2 (widespread fluorescence) (Supplementary Table S2).

For inferential analysis, fluorescence grades were additionally dichotomized into a binary endpoint: fluorescence-negative (Grade 0) and fluorescence-positive (Grades 1–2).

The primary clinical outcome was the change in fluorescence status from baseline to the end of the CAP intervention period. Secondary observational outcomes included qualitative assessment of wound appearance, exudate characteristics, and patient tolerance of the treatment. No treatment-related adverse events or evidence of tissue damage were observed during the study period, and treatment was well tolerated.

### 2.5. Statistical Analysis

This study comprised three different components: in vitro, porcine, and clinical service evaluation, and does not represent a planned prospective randomized study with predefined hypothesis testing. An a priori sample size determination was not necessary. Data distribution and variance assumptions were assessed prior to inferential testing using standard statistical methods. Statistical analyses were performed using R (version 4.1.2) and SQL (SQL Server Management Studio 17). When assumptions for parametric testing were not met, nonparametric methods were applied.

#### 2.5.1. In Vitro Model

Colony-forming unit (CFU) counts were recorded for each organism and CAP exposure duration. Results are presented descriptively using individual replicate points and mean values. Log10 reductions were calculated relative to untreated controls at the corresponding time point (log10[CFUcontrol/CFUtreatment]).

#### 2.5.2. Porcine Model

Microbiological outcomes were expressed as log10 CFU per gram of tissue. Histology outcomes were analyzed as continuous (e.g., percent re-epithelialization, epithelial thickness) or semi-quantitative ordinal scores, as appropriate. For Animal #2, wound-level change scores were calculated as Day 11 minus Day 4 (diff d11-d4) for each endpoint. Between-group differences in change scores were evaluated across treatment duration (2 min, 4 min, untreated) and across frequency clusters (2×/week, 3×/week, 5×/week) using nonparametric methods. Post hoc pairwise Wilcoxon rank-sum tests (exact or continuity-corrected) were performed with Bonferroni adjustment for multiple comparisons. Effect sizes were calculated by pooling across frequency regimes, using Hodges–Lehmann (median difference) with 95% CIs obtained via nonparametric bootstrapping. Two-sided *p*-values < 0.05 were considered statistically significant. Any wounds that appeared to be disturbed or injured during the study would have been excluded from analysis; however, no cases were observed.

#### 2.5.3. Clinical Model

Fluorescence grades (0–2) were summarized at each measurement point (MP1–MP5), corresponding to weeks 5–9 of the treatment period, using descriptive statistics. Group differences were evaluated using one-way ANOVA with Bonferroni-adjusted post hoc comparisons, with complementary nonparametric analyses performed. Changes in dichotomized fluorescence status were assessed using McNemar’s test and exact (Clopper-Pearson) 95% CI. Twenty ulcers were treated; wounds with missing or uninterpretable fluorescence grades at any measurement point were excluded from complete-case longitudinal analyses. All tests were two-sided with α = 0.05.

## 3. Results

### 3.1. In Vitro Antimicrobial Testing

**Figure 2:**
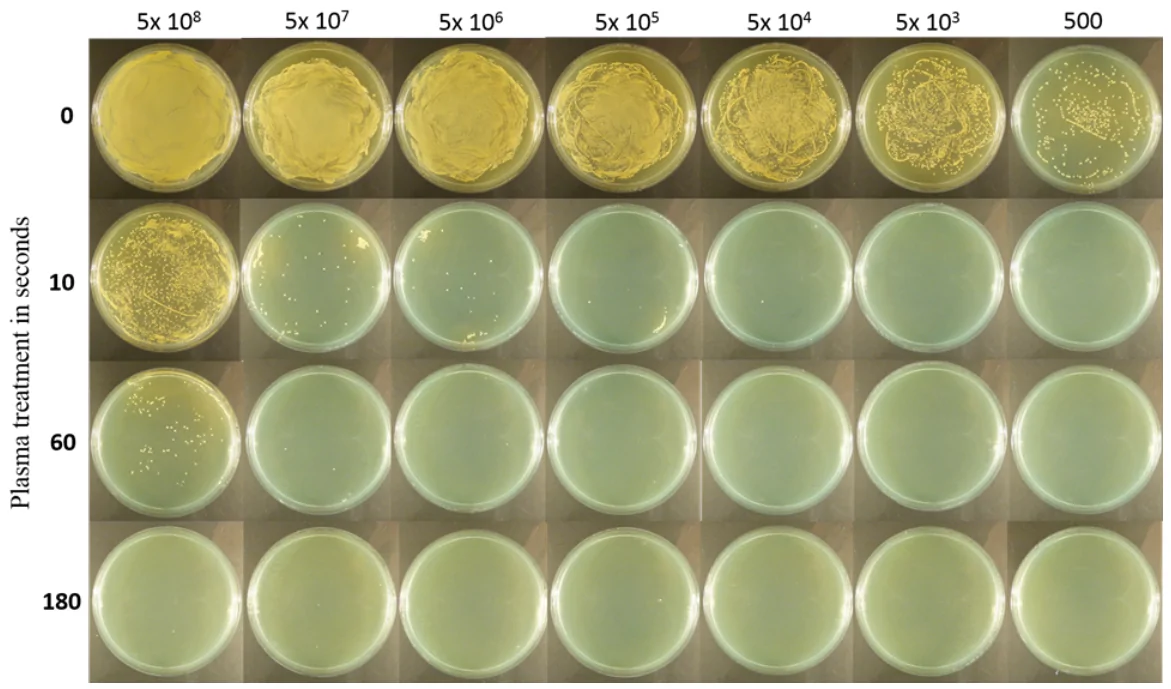
Representative image demonstrating the antimicrobial effect of cold atmospheric plasma.

Exposure of bacterial cultures to cold atmospheric plasma (CAP) resulted in a marked reduction in viable colony-forming units (CFUs) across all tested wound-relevant pathogens. Figure 2 shows agar plates inoculated with decreasing MRSA bacterial loads from 500,000,000 ($5\times 10^{8}$) to 500 ($5\times 10^{2}$) cells and exposed to plasma for 0, 10, 60, or 180 s. At baseline, dense bacterial growth was present across all inoculum levels. Following a 10-s CAP exposure, a substantial decrease in colony density was observed across all inoculum levels. Near-complete sterilization was achieved with exposure durations of 60 to 180 s.

**Figure 3:**
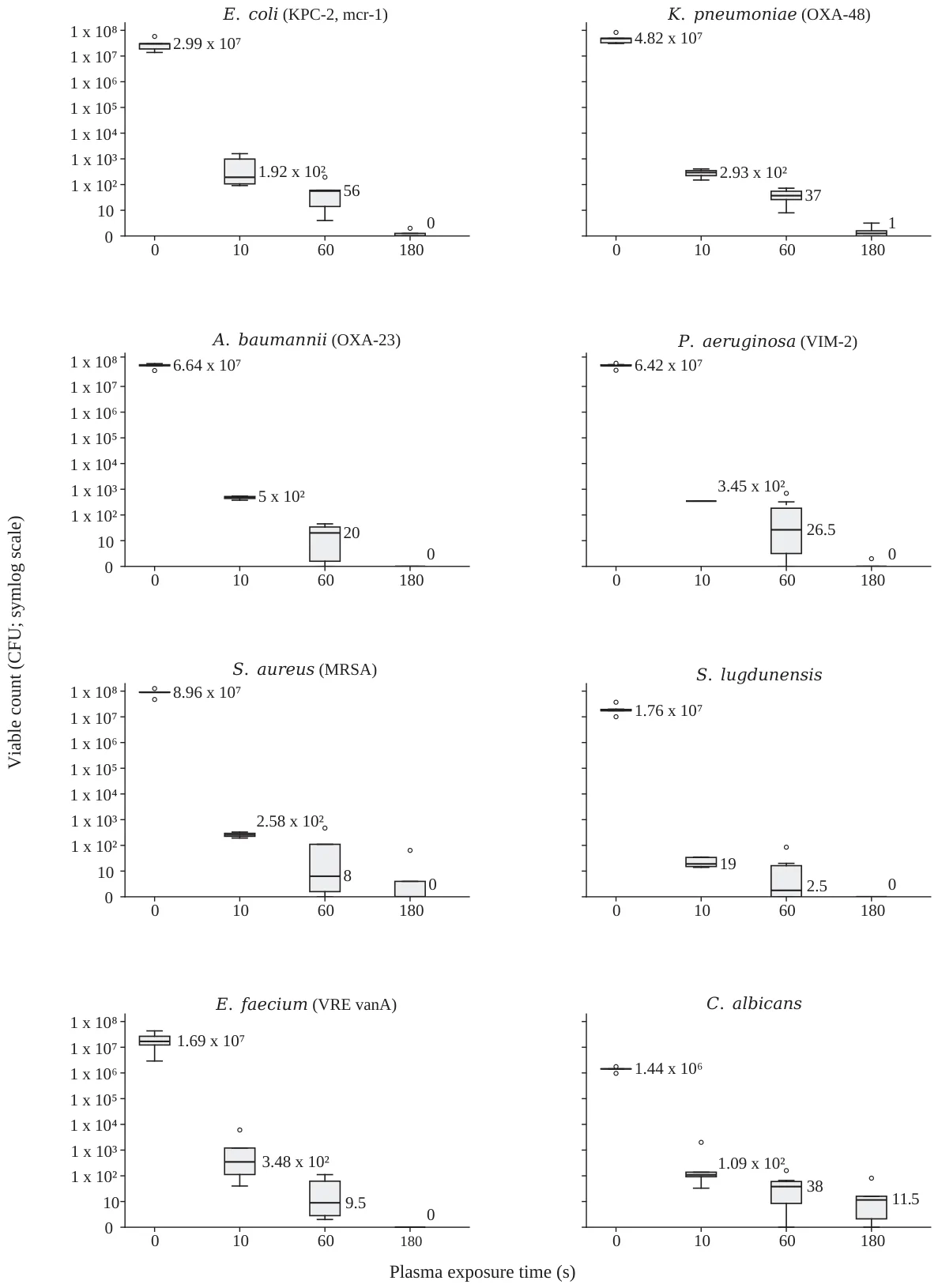
Colony-forming unit (CFU) counts for eight clinically relevant wound pathogens following exposure to cold atmospheric plasma (CAP) for 0, 10, 60, or 180 s. Box plots display the median (central line), interquartile range (25th–75th percentiles), and data range. Individual points represent independent replicates. A time-dependent reduction in CFU counts was observed with increasing CAP exposure duration.

Quantitative CFU analyses for eight clinically relevant wound pathogens are presented in Figure 3. All organisms exhibited a consistent inverse correlation between CAP exposure time and CFU recovery. Baseline CFU counts ranged from approximately $1.4\times 10^{6}$ for *Candida albicans* to $8.9\times 10^{7}$ for *Staphylococcus aureus* (Figure 3). After 10 s of CAP treatment, CFU counts declined sharply, ranging from 38 to 1084 across species. At 60 s, CFU values further decreased to a range of 7.7 to 77, and by 180 s, residual CFUs were minimal, ranging from 0 to 20. The antimicrobial effect of the CAP was tested in five independent replicates for each species. Exposure-dependent log-reduction trajectories across all tested organisms are shown in Supplementary Figure S1. These findings confirm the rapid and broad-spectrum antimicrobial efficacy of CAP, including activity against high-burden and biofilm-forming pathogens.

### 3.2. Porcine MRSA-Infected Wound Model

#### 3.2.1. Microbiological Results

**Figure 4:**
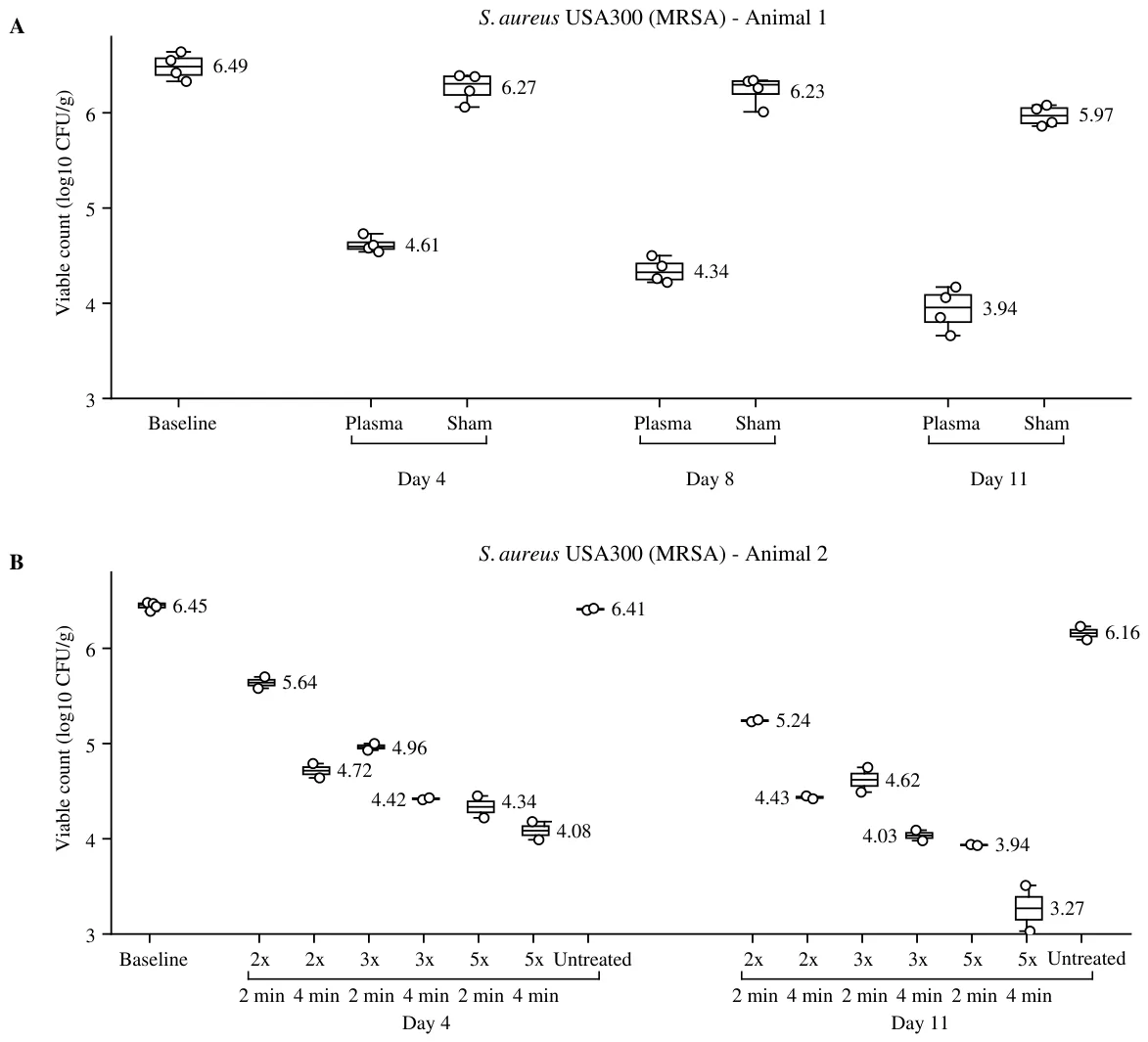
MRSA USA300 Bacterial counts following cold atmospheric plasma (CAP) treatment—(**A**) Animal #1 and (**B**) Animal #2.

In the MRSA-infected deep dermal porcine model, CAP-treated wounds showed lower bacterial counts than sham-treated wounds at all assessed time points. CAP treatments were administered on Days 0, 4, and 8, resulting in one, two, and three cumulative treatments prior to the Day 4, 8, and 11 assessments, respectively. (CAP, 4.61 ± 0.08, 4.34 ± 0.13, and 3.94 ± 0.23 Log CFU/g on Days 4, 8, and 11, respectively; Sham, 6.27 ± 0.16, 6.23 ± 0.16, and 5.97 ± 0.11 Log CFU/g on Days 4, 8, and 11, respectively). Compared with Sham treatments, CAP-treated wounds showed reductions of 1.65 (95% CI 1.42–1.90), 1.89 (95% CI 1.64–2.14), and 2.04 (95% CI 1.69–2.37) log units on Days 4, 8, and 11, respectively, equivalent to 97.8–99.1% decreases (corresponding to 46×, 78×, and 107× lower CFU/g in CAP vs. sham), whereas sham-treated wounds maintained persistently elevated bacterial levels. Individual wound data and group mean for each time point are presented in Figure 4.

In the second animal, CAP-treated wounds exhibited decreasing MRSA CFU counts from Day 4 to Day 11 in a dose-dependent manner, while untreated control wounds showed consistently high bacterial loads across both time points. Across both Day 4 and Day 11, all plasma treatment groups and frequencies showed reductions in CFU; however, wounds treated with the plasma dressing for 4 min consistently showed lower bacterial counts than those treated for 2 min, demonstrating a clear treatment-time effect. The greatest reductions were seen in wounds treated five times, reaching 99.4% (2 min) and 99.87% (4 min) reductions compared with untreated controls, and 99.69% and 99.93% reductions relative to baseline, respectively. These results are shown in Figure 4.

In Animal #1 (plasma vs. sham), by Day 11, plasma-treated wounds showed substantially lower MRSA burden than sham (mean 3.94 vs. 5.97 log10 CFU/g; difference = 2.03 log10; *p* < $2\times 10^{-16}$), consistent with an increasing treatment effect over time. In Animal #2 (regimens vs. untreated), several CAP regimens achieved statistically significant reductions in MRSA versus untreated controls. By Day 11, all evaluated CAP regimens showed significant MRSA reductions versus untreated controls (*p* < 0.001), with mean MRSA decreasing from 6.16 log10 CFU/g in untreated wounds to as low as 3.27 log10 CFU/g in the 4-min, 5×/week regimen (Supplementary Figure S16).

#### 3.2.2. Re-Epithelialization and Epithelial Thickness

A descriptive histological comparison was made between plasma and sham wound dressings on days 4, 8 and 11. There was comparable re-epithelialization and epithelial thickness between groups, suggesting that plasma treatment had no deleterious effects on wound re-epithelialization (Supplementary Figures S2 and S3). Pilot animal #2 received multiple cold plasma treatments for 2 or 4 min, and wounds were collected on Days 4 and 11 (n = 2 replicates per treatment/assessment time point). Repeated plasma applications (3–5×) and longer treatment duration (4 min vs. 2 min) synergistically enhanced wound re-epithelialization by Day 11 in a dose-dependent manner, with the most robust closure seen in the 4-min, 5× group (Supplementary Figures S8 and S9).

Quantitative assessment of Day 11-Day 4 change scores showed significantly greater improvement in percent re-epithelialization in wounds treated with 4-min CAP compared with 2-min CAP (Hodges–Lehmann difference: +31.2 percentage points; 95% CI: +7.8 to +45.2; Bonferroni-adjusted Wilcoxon rank-sum *p* = 0.045). In contrast, change in epithelial thickness did not differ between 4-min and 2-min treatments after adjustment (*p* = 0.93), indicating no evidence of abnormal epithelial hyperproliferation.

#### 3.2.3. White Cell Infiltration & Granulation Tissue Formation

Across both animals, plasma treatment did not exacerbate inflammation and demonstrated a trend toward reduced white cell infiltration (WCI) (Supplementary Figures S6, S7, S11 and S12), particularly with longer-duration (4 min) and higher-frequency (3–5×) treatments, suggesting a possible anti-inflammatory effect that supports tissue repair.

Plasma-treated wounds displayed slightly higher or comparable scores to untreated controls depending on treatment duration and frequency and by Day 11 tissue formation was evident in all groups; however, 4-min plasma groups maintained higher granulation tissue compared to 2-minute plasma and controls, suggesting that longer-duration, repeated plasma applications may sustain fibroblast and extracellular matrix activity longer into the repair process.

Plasma-treated wounds consistently demonstrated greater angiogenesis than untreated control wounds, especially at early and intermediate time points. On Day 4, plasma-treated groups already showed a measurable increase in angiogenesis relative to controls, and by Day 11, this difference persisted, with plasma-treated wounds maintaining higher vessel density scores (Supplementary Figure S10).

#### 3.2.4. Tissue Necrosis, Fibrosis, and Fatty Infiltration

Fibrosis scores, representing collagen deposition and extracellular matrix remodeling, increased over time in both untreated controls and plasma-treated wounds. However, by Day 11, plasma-treated wounds achieved higher scores than controls, suggesting that plasma treatment may accelerate collagen deposition and matrix organization, thereby promoting more rapid dermal strengthening (Supplementary Figures S4 and S13).

Pooling across frequency regimes (2×, 3×, and 5× per week), wound-level change scores (Day 11–Day 4) demonstrated greater improvement with 4-minute group than 2-minute CAP for re-epithelialization (Hodges–Lehmann difference: +31.2%; 95% CI: +7.8% to +45.2%; adjusted *p* = 0.045), fatty infiltration (difference: −1.0; 95% CI: −2.0 to −1.0; adjusted *p* = 0.026), and fibrosis score (difference: −1.0; 95% CI: −2.0 to −1.0; adjusted *p* = 0.029).

Tissue necrosis was initially present in all groups at Day 4. Plasma-treated wounds, however, showed a sharper decline in necrosis scores and almost complete resolution by Day 11. In contrast, untreated control wounds retained higher levels of necrosis at comparable time points. Masson’s trichrome staining further indicated that plasma treatment was associated with increased angiogenesis, reduced necrosis, and modestly higher fibrosis, consistent with enhanced tissue remodeling. Fatty infiltration remained minimal across both groups, suggesting that plasma primarily influenced vascularization, tissue preservation, and extracellular matrix deposition rather than adipocyte accumulation (Supplementary Figures S14 and S15).

#### 3.2.5. Immune Cells

A similar inflammatory response observed in Animal #1 was also seen in Animal #2, where plasma treatment shaped the immune cell profile toward a more efficient inflammatory-to-repair transition, with reduced polymorphonuclear neutrophil (PMN) persistence, maintenance of lymphocyte and macrophage activity, stable mast cell presence, and no excess giant cell formation. Collectively, this immune cell balance suggests plasma therapy promotes controlled inflammation and supports regenerative processes in the wound microenvironment (Supplementary Figure S5).

Overall, descriptive histological evaluations showed that plasma-treated wounds in both animals displayed preserved or improved re-epithelialization, earlier formation of new blood vessels, and more rapid resolution of inflammatory infiltrates compared with controls. These qualitative observations were consistent with the quantitative findings but are limited by the small sample size inherent to this pilot study design. Polymorphonuclear cell infiltration was reduced in treated wounds, while vascular density and granulation tissue formation were increased. Surface bacterial aggregates were less prominent in CAP-treated wounds relative to controls.

### 3.3. Clinical Fluorescence Outcomes: Suppression of Wound Bioburden

Twenty chronic arterial and venous ulcers were treated with CAP therapy twice weekly for five weeks. Fluorescence imaging with the MolecuLight^®^ device demonstrated a progressive reduction in bacterial signal intensity across the intervention period. Three ulcers had missing or uninterpretable fluorescence grades at one or more measurement points and were excluded from the complete-case longitudinal grade analysis (Supplementary Table S9). Almost all wounds presented with strong red fluorescence at baseline, consistent with clinically significant bioburden (>$10^{4}$ CFU/g). Among ulcers with interpretable fluorescence grades at all five measurement points (MP) (n = 17), fluorescence-negative wounds (Grade 0) increased from 0/17 (0.0%) at MP1 (Week 5) to 12/17 (70.6%) at MP5 (exact 95% CI: 44.0% to 89.7%, Clopper-Pearson).

**Figure 5:**
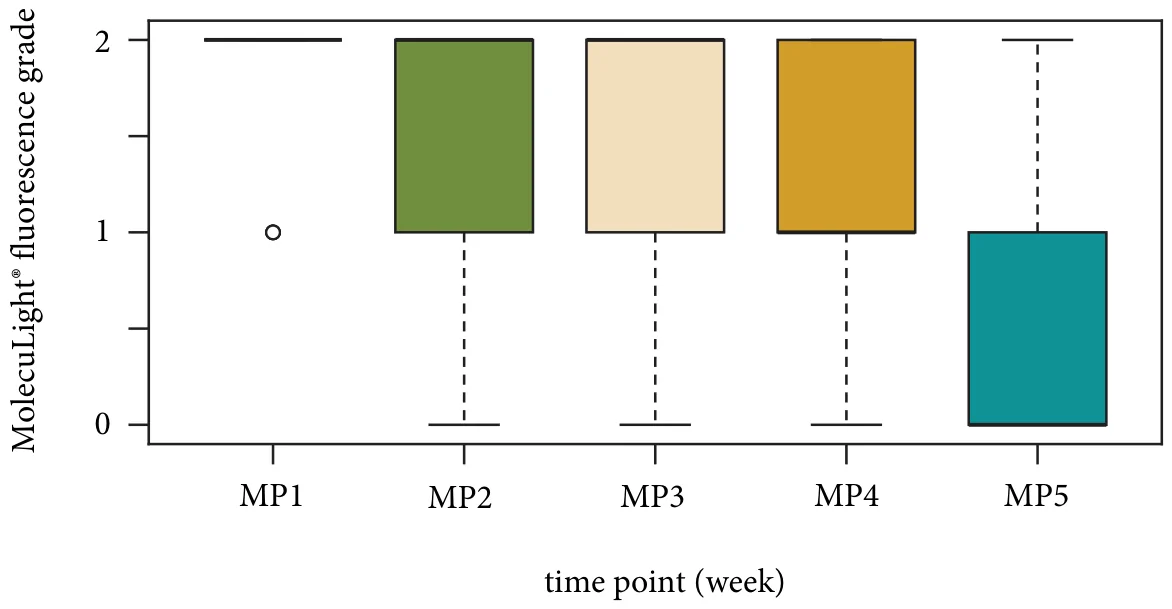
Distribution of MolecuLight^®^ fluorescence grades across five measurement points (MP1–MP5) during CAP therapy. Box plots display the median (central line), interquartile range (25th–75th percentiles), and data range. A progressive decline in fluorescence grades was observed over time. Statistical analysis using one-way ANOVA demonstrated a significant overall effect of time on fluorescence grades (*p* = 4.08 × 10^−7^).

Across the five measurement points MP1 to MP5 (Weeks 5–9), a clear downward shift in fluorescence grade was observed (Figure 5 and Supplementary Table S8). Grade 2 fluorescence predominated at baseline MP1 (Week 5), (13/17, 76.5%), and by MP5 (Week 9), most wounds were Grade 0 (12/17, 70.6%) and only 1/17 (5.9%) remained in Grade 2. Overall, 16/17 (94.1%) wounds were Grade 0 or 1 at MP5 (Week 9). This trend was statistically significant when fluorescence grades were compared across time points (one-way ANOVA, *p* < 0.001) (Table 1). Detailed wound-level fluorescence grades are provided in Supplementary Table S8.

Importantly, no treatment-related adverse events or evidence of tissue damage were observed, and treatment was well tolerated. These findings confirm CAP’s ability to produce clinically measurable antibacterial effects, consistent with in vitro and porcine outcomes.

## 4. Discussion

Cold atmospheric plasma is increasingly investigated as both an antimicrobial and a wound-modulatory technology. In chronic wounds, persistent biofilm colonisation, excessive inflammation, and impaired cellular responses collectively delay healing [10]. Further, wound infections are the leading cause of morbidity and mortality in acute burn wounds [45]. Rather than acting solely as a surface disinfectant, CAP appears to influence multiple components of the wound microenvironment, including microbial burden, inflammatory signaling, and tissue repair processes [46].

The goal of the study was to integrate in vitro antimicrobial kinetics, a porcine MRSA biofilm model, and clinical fluorescence imaging to evaluate CAP across mechanistic, preclinical, and clinical contexts using a single large area CPT-based device. Based on assumptions derived from previous studies’ results, we hypothesized that CAP would favourably disrupt biofilm and guide the inflammatory response without inducing apoptosis. Taken together, the findings indicate that CAP consistently reduces bacterial burden and is associated with reproducible tissue-level responses relevant to wound healing.

Cold plasma’s antimicrobial activity is primarily driven by reactive oxygen and nitrogen species (RONS), which act synergistically to destabilize microbial membranes, alter protein structure, and fragment DNA [27,29]. Unlike antibiotics, CAP does not rely on enzyme targets that are vulnerable to bacterial genetic resistance. Moreover, CAP’s effect is not limited to bacteria, as it has demonstrated potent antiviral activity by oxidizing viral capsid proteins, disrupting lipid envelopes, and damaging viral nucleic acids, leading to loss of infectivity. These effects have been reported across multiple viral families, supporting broader antimicrobial activity beyond bacteria [47,48,49,50,51].

In chronic wounds, these mechanistic properties provide a rationale for the use of large-area CAP.

The porcine MRSA biofilm model bridges the gap between planktonic in vitro conditions and the complexity of human wounds. Unlike agar-based assays, this model incorporates extracellular matrix, host cells, inflammatory responses, and vascularisation, all of which influence antimicrobial efficacy.

Quantitatively, large area CAP reduced MRSA burden by 1.65, 1.89, and 2.04 log10 CFU/g versus sham on Days 4, 8, and 11, equivalent to 97.8%, 98.7%, and 99.1% reductions. In Animal #2, the strongest 5×/week regimens achieved 2.22 log10 (99.4%) and 2.89 log10 (99.87%) reductions versus untreated controls, with corresponding reductions of 2.51 log10 (99.69%) and 3.18 log10 (99.93%) relative to baseline.

When interpreted alongside published porcine wound-biofilm studies evaluating PHMB irrigation, silver gelling-fibre dressings, PHMB-containing collagen matrices, and multimodal wound matrices [52,53,54,55,56,57], the CPT system achieved bacterial reductions within the upper reported efficacy range, including 97.8–99.1% reduction versus sham in the 24-h MRSA biofilm model and up to 99.87% reduction versus untreated controls in the 72-h MRSA biofilm model, while preserving tissue compatibility.

From a safety and healing perspective, histological analysis showed that 4-min treatments were associated with improved resolution of fatty infiltrate and a smaller increase in fibrosis compared with 2-min treatments, while epithelial thickness and other inflammatory parameters did not differ significantly between treatment durations. In addition, CAP-treated wounds exhibited preserved or improved re-epithelialization, earlier angiogenesis, and faster resolution of inflammatory infiltrates. Previous evidence suggests that CAP increases angiogenic molecular activity, including upregulation of endothelin-1 (ET-1), fibroblast growth factor-2 (FGF-2), epidermal growth factor (EGF), vascular endothelial growth factor (VEGF), transforming growth factor (TGF), and angiogenin (ANG) [36,37,38]. These findings differentiate CAP from several conventional topical antiseptics, such as povidone iodine or chlorhexidine, which can impair fibroblast and keratinocyte activity at bactericidal concentrations [58,59,60]. The porcine data therefore suggest that CAP occupies a favourable position in the antimicrobial spectrum, combining efficacy with tissue compatibility.

MolecuLight^®^ fluorescence imaging was used as a semi-quantitative surrogate marker of surface bacterial burden rather than a direct measure of microbial load. Red fluorescence correlates with bacterial concentrations above approximately $10^{4}$ CFU/g of tissue but does not provide precise quantification or species identification.

The observed shift from positive to negative fluorescence over the treatment period, therefore, reflects a reduction in clinically relevant surface bioburden rather than an exact log reduction; Grade 2 predominated at MP1 (13/17, 76.5%), whereas by MP5, 16/17 wounds (94.1%) were Grade 0 or 1 and 12/17 (70.6%) were Grade 0.

Existing literature suggests that lower surface bioburden is associated with improved wound bed conditions, reduced inflammation, and enhanced responsiveness to advanced therapies [61,62]. The present findings align with this broader evidence base, but the relation between fluorescence reduction and healing requires further controlled trials.

Across all three experimental levels, a dose-dependent pattern emerged. In vitro, longer exposure times produced greater log CFU reductions. In the porcine model, 4-min treatments administered five times per week led to more pronounced reductions in MRSA burden and more favourable histological changes than 2-min treatments. Clinically, repeated twice-weekly applications over five weeks were associated with progressive decreases in fluorescence.

These findings suggest the optimal balance between exposure duration, treatment frequency, and clinical feasibility likely varies depending on wound type, depth, and microbial burden.

The data support a differentiated indication profile for CAP. In biofilm-rich chronic wounds, such as venous leg, pressure, and diabetic foot ulcers, CAP appears particularly suited as an adjunct to reduce surface bioburden and modulate inflammation. Of importance, chronic wounds are often hypoxic, and CAP is known to produce exogenous NO, which is known to be anti-inflammatory and angiogenic [63].

CAP is equally promising in the acute wound, where bacterial burden increases morbidity and mortality. In burn care, where infection control and tissue preservation are critical, existing CAP-based plasma studies have demonstrated antimicrobial effects without thermal damage, supporting potential applications in partial-thickness burns or graft donor sites [26,45,64]. Oliver et al. [45] demonstrated that CAP significantly reduced bacterial burden and augmented wound healing in deep partial and full-thickness burns compared to controls. Interestingly, large-area CAP could be a therapeutic target for treating surgical site infections (SSIs). Reports suggest that as many as 3 out of 10 surgeries are associated with SSIs, leading to increased hospitalization stays and healthcare costs [65].

The findings support the concept of using CAP primarily as a surface decontamination tool, well-suited to reducing bioburden during the phases of wound healing, around dressing changes, or during preparation for advanced therapies.

## 5. Conclusions

Across in vitro testing, porcine MRSA biofilm wound models, and clinical patient evaluation using fluorescence imaging, the CPTpatch^®^/CPTcube^®^ cold atmospheric plasma system produced reproducible antimicrobial effects, dose-dependent reductions in bacterial burden, and decreased bacterial fluorescence, while demonstrating favorable tissue compatibility. The antimicrobial panel included clinically relevant bacterial and fungal wound pathogens carrying resistance determinants such as blaKPC, blaOXA, vanA, and mecA, and the porcine wound model used MRSA USA-300 biofilm, directly supporting CAP activity against resistance-relevant wound pathogens. In the porcine model, bacterial reductions of 97.8–99.1% versus sham and up to 99.87% versus untreated controls place the CPT system within the upper reported efficacy range of comparable porcine wound-biofilm interventions. The standardized sealed-patch exposure and controlled treatment field may help explain the pronounced antimicrobial effects observed with the CPT system compared with more heterogeneous CAP modalities. Together, these findings position the CPT system as a standardized, resistance-relevant, non-antibiotic, large-area CAP platform that may complement existing wound-care strategies, particularly in biofilm-rich chronic wounds and potentially in larger thermal-injury settings requiring dedicated protocols.

## Figures and Tables

**Table 1:** Distribution of fluorescence grades across measurement points.

Time Point	N	Grade 0	Grade 1	Grade 2	Mean	Median (Q1–Q3)	SD
MP1 (Week 5)	17	0 (0.0%)	4 (23.5%)	13 (76.5%)	1.76	2 (2–2)	0.44
MP2 (Week 6)	17	3 (17.6%)	4 (23.5%)	10 (58.8%)	1.41	2 (1–2)	0.80
MP3 (Week 7)	17	1 (5.9%)	7 (41.2%)	9 (52.9%)	1.47	2 (1–2)	0.62
MP4 (Week 8)	17	4 (23.5%)	6 (35.3%)	7 (41.2%)	1.18	1 (1–2)	0.81
MP5 (Week 9)	17	12 (70.6%)	4 (23.5%)	1 (5.9%)	0.35	0 (0–1)	0.61

## Data Availability

The data presented in this study are stored on an institutional server. The data are not publicly available due to privacy and ethical restrictions in accordance with applicable data protection regulations and institutional requirements.

## Supplementary Materials

The following supporting information can be downloaded at https://www.mdpi.com/article/10.3390/biomedicines14081750/s1, Supplementary Methods. Table S1: Semi-quantitative inflammatory cell infiltrate scoring system; Table S2: Fluorescence and histological scoring criteria; Table S3: Baseline patient characteristics; Table S4: Baseline wound characteristics and fluorescence dataset availability; Table S5: De-identified wound-level baseline data and MolecuLight^®^ dataset completeness; Table S6: Concomitant standard wound care protocol; Table S7: Observed dressing and supportive care exposure during the CAP phase; Table S8: Raw analyzable MolecuLight^®^ fluorescence grades; Table S9: MolecuLight^®^ fluorescence series excluded from repeated-measures analysis and reasons for exclusion; Figure S1: Log reduction in colony-forming units (CFUs) across all tested organisms at increasing plasma exposure times; Figure S2: Percentage re-epithelialization in plasma-treated versus sham-treated wounds in Animal #1; Figure S3: Epithelial thickness (µm) in plasma-treated versus sham-treated wounds in Animal #1; Figure S4: Fibrosis, angiogenesis, necrosis, and fatty infiltration scores in plasma-treated versus sham-treated wounds in Animal #1; Figure S5: PMN, lymphocyte, macrophage, mast cell, and giant cell scores in plasma-treated versus sham-treated wounds in Animal #1; Figure S6: White cell infiltration scores between plasma and sham dressings—in Animal #1; Figure S7: Granulation formation scores between plasma and sham dressings—in Animal #1; Figure S8: Percentage re-epithelialization between controls and plasma treated wounds at day 4 and 11 in Animal #2; Figure S9: Epithelial thickness (µm) between controls and plasma treated wounds at day 4 and 11 in Animal #2; Figure S10: Angiogenesis scores in control and plasma-treated wounds at Days 4 and 11 in Animal #2; Figure S11: White cell infiltration scores in control and plasma-treated wounds at Days 4 and 11 in Animal #2; Figure S12: Granulation tissue scores in control and plasma-treated wounds at Days 4 and 11 in Animal #2; Figure S13: Fibrosis scores in control and plasma-treated wounds at Days 4 and 11 in Animal #2; Figure S14: Necrosis tissue scores between controls and plasma treated wounds at day 4 and 11 in Animal #2; Figure S15: Fatty infiltration scores in control and plasma-treated wounds at Days 4 and 11 in Animal #2; Figure S16: MRSA Reduction Across Treatment Regimens following Cold Atmospheric Plasma (CAP) treatment.

## Abbreviations

The following abbreviations are used in this manuscript:

ANG	Angiogenin
ANOVA	Analysis of variance
CAP	Cold atmospheric plasma
CFU	Colony-forming units
CPT	Cold plasma therapy
DBD	Dielectric barrier discharge
EGF	Epidermal growth factor
ET-1	Endothelin-1
FGF-2	Fibroblast growth factor-2
IACUC	Institutional Animal Care and Use Committee
MRSA	Methicillin-resistant *Staphylococcus aureus*
OD_600_	Optical density at 600 nm
PMN	Polymorphonuclear neutrophils
RONS	Reactive oxygen and nitrogen species
SSI	Surgical site infection
TGF	Transforming growth factor
VEGF	Vascular endothelial growth factor
WCI	White cell infiltration
