# Supplementary material for: Cold Atmospheric Plasma Disrupts Microbial Wound Bioburden: In Vitro, Porcine MRSA Biofilm, and Clinical Fluorescence Evidence from Bench to Bedside
Source: Biomedicines. 2026 Aug 3;14(8):1750. doi: 10.3390/biomedicines14081750 (PMC13510128; doi:10.3390/biomedicines14081750)
Supplement: Supplementary file 1 [file biomedicines-14-01750-s001.zip › biomedicines-4296546-supplementary.pdf]

Methods

Concomitant Standard Wound Care: Across all study phases, patients continued to receive protocol-concordant standard wound care. This comprised wound cleansing with locally used agents, including polyhexanide (PHMB) with betaine surfactant (Prontosan®), octenidine/allantoin wipes (Octenilin®), and povidone-iodine solution (Videne®), together with superficial debridement when clinically indicated and application of non-occlusive dressings selected according to wound condition and local practice. During the CAP phase, the same standard wound care was delivered before and after each plasma application, with off-loading and supportive care added where clinically indicated. No concurrent topical antibiotics were introduced during the intervention phase.

MolecuLight® Analytic Cohort and Exclusions: MolecuLight fluorescence outcomes were analyzed in the 17 wounds with complete, interpretable ordinal fluorescence series retained in the final grading dataset. Three wound series were excluded from repeated-measures analysis. One wound (W16 / study wound 416) had an incomplete five-visit series due to a missing or non-gradable fluorescence image. Two additional wound series (W03 / study wound 403 and W18 / study wound 418) were recorded as imaged across weeks 5–9; however, no finalized ordinal fluorescence grades were retained following image review. These cases were therefore classified as excluded due to non-standardized or uninterpretable acquisition conditions (e.g., inconsistent lighting, background conditions, or camera angle), rather than true clinical missingness.

Clinical Service-Evaluation Context and Concomitant Care Exposure: This study was conducted as part of a broader service-evaluation project comprising a 4-week standard wound care control phase (weeks 1–4), a 5-week CAP intervention phase (weeks 5–9), and a follow-up phase without CAP (weeks 10–13). The present analysis focuses on fluorescence outcomes during the intervention phase. Observed CAP-phase concomitant care included silver-based dressings in 11/20 wounds, iodine-based dressings in 3/20, neutral/non-antimicrobial dressings in 6/20, hydrogel in 1/20, and topical steroid adjunct use in 7/20 wounds.

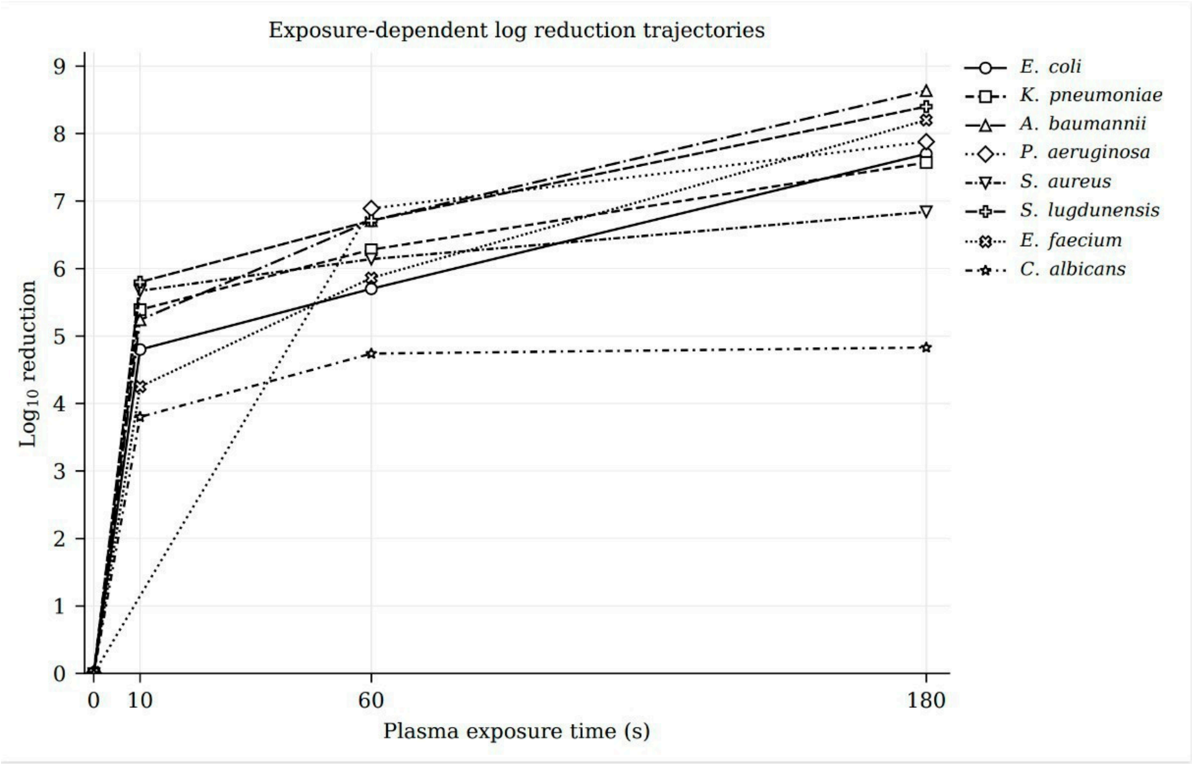

Supplementary Figure S1: Log reduction in colony-forming units (CFU) across all tested organisms at increasing plasma exposure times

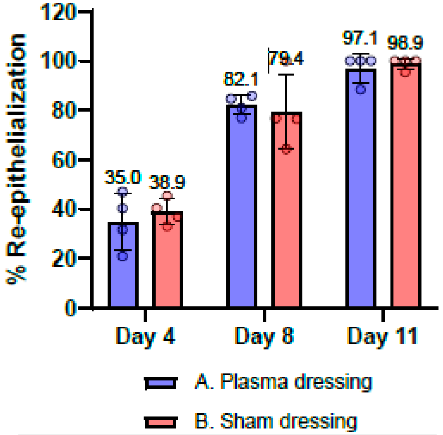

Supplementary Figure S2. Percentage Re-epithelization between plasma and sham dressings -in Animal #1.

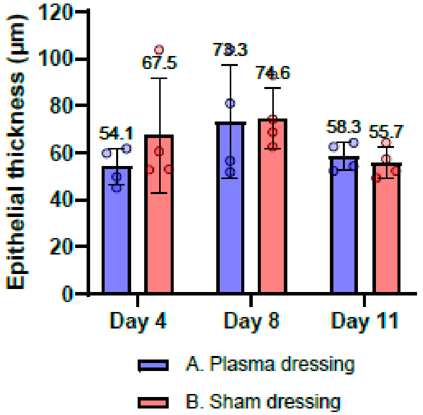

Supplementary Figure S3: Epithelial thickness (μm) between plasma and sham dressings -in Animal #1.

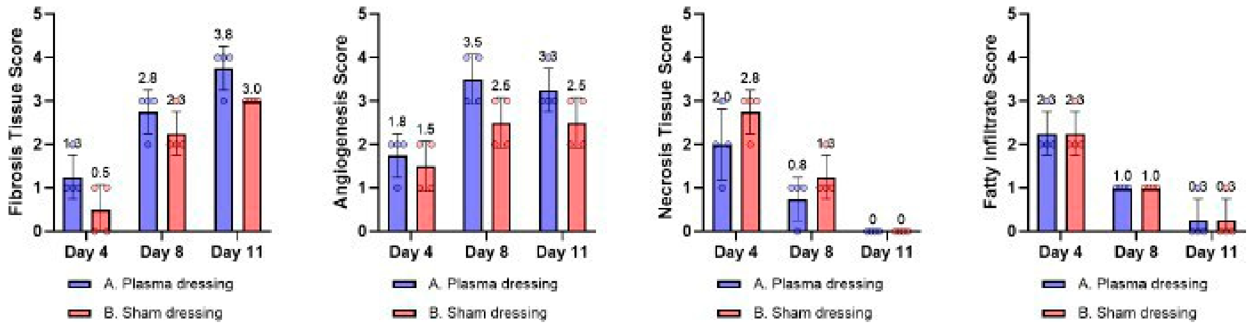

Supplementary Figure S4. Left to right – Fibrosis, angiogenesis, necrosis, and fatty infiltration scores between plasma and sham dressing on Animal #1.

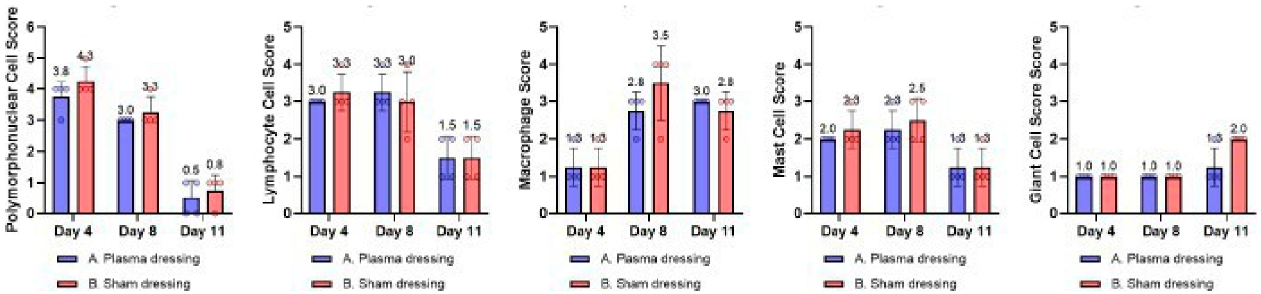

Supplementary Figure S5. Left to right – PMN, Lymphocyte, Macrophage, Mast, and Giant cell scores between plasma and sham dressings in Animal #1.

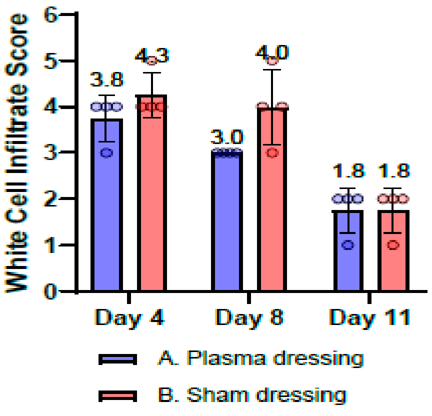

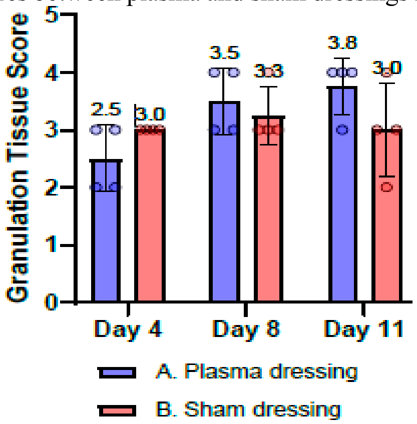

Supplementary Figure S6: White cell infiltration scores between plasma and sham dressings -in Animal #1

Supplementary Figure S7: Granulation formation scores between plasma and sham dressings -in Animal #1

.

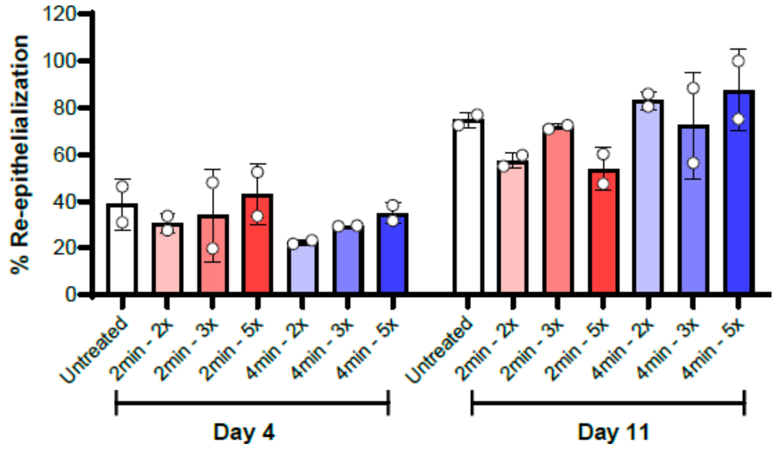

Supplementary Figure S8. Percentage re-epithelialization between controls and plasma treated wounds at day 4 and 11 in Animal #2.

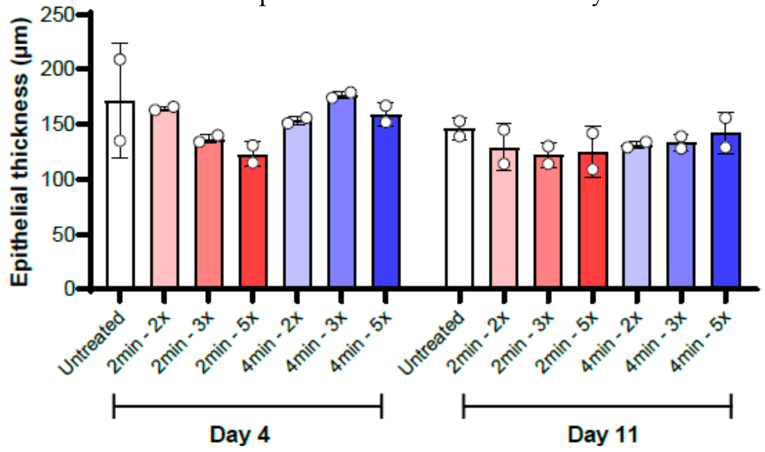

Supplementary Figure S9. Epithelial thickness (µm) between controls and plasma treated wounds at day 4 and 11 in animal #2.

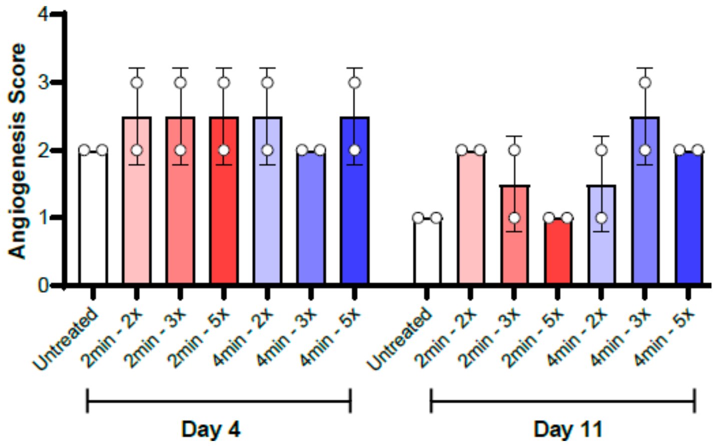

Supplementary Figure S10. Angiogenesis scores in control and plasma-treated wounds at Days 4 and 11 in Animal #2

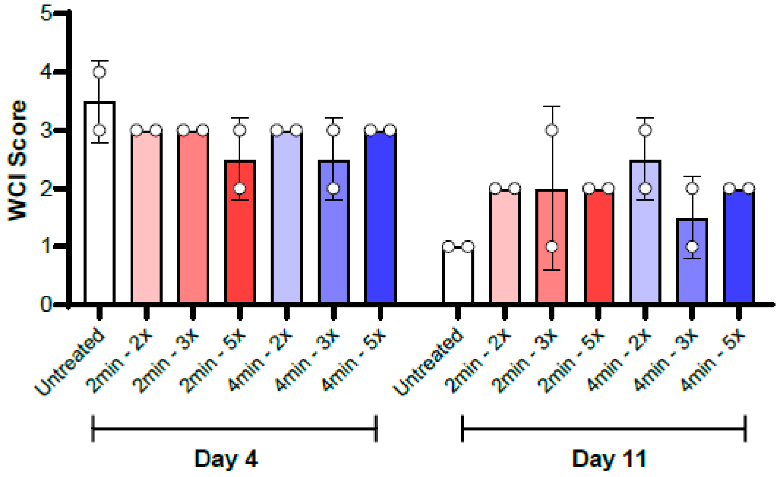

Supplementary Figure S11. WCI scores between controls and plasma treated wounds in Animal #2

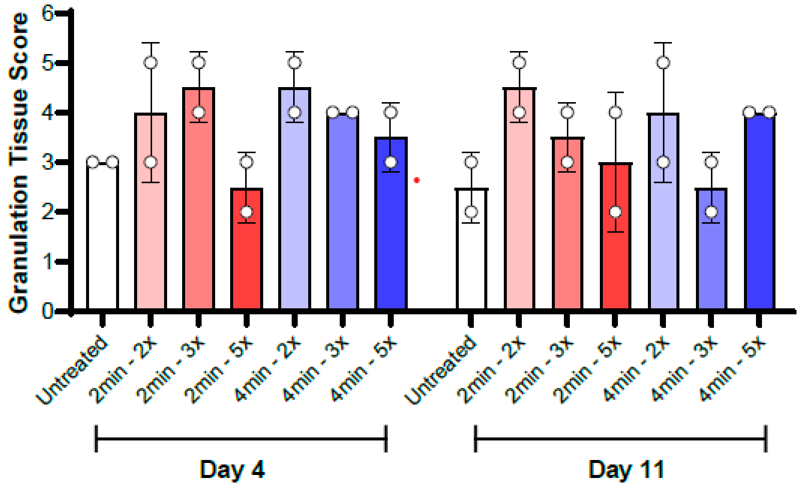

Supplementary Figure S12: Granulation tissue scores between controls and plasma treated groups at day 4 and 11 in Animal #2.

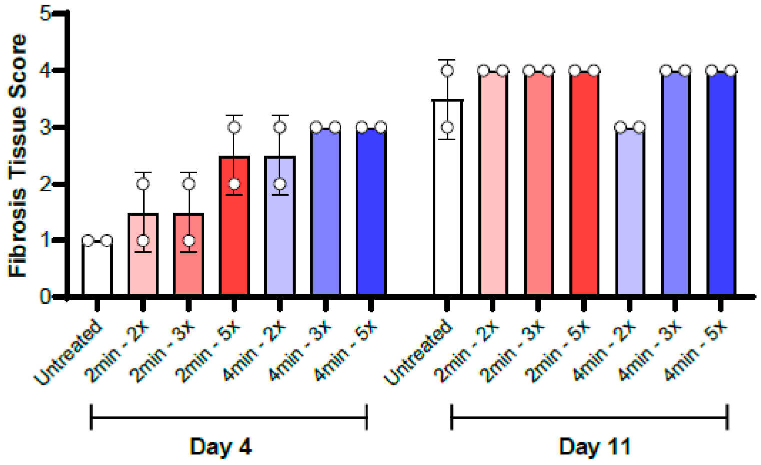

Supplementary Figure S13. Fibrosis scores in control and plasma-treated wounds at Days 4 and 11 in Animal #2.

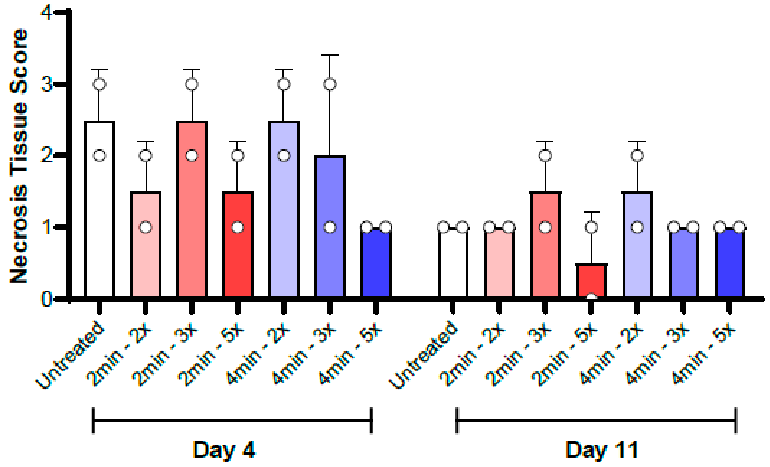

Supplementary Figure S14: Necrosis tissue scores between controls and plasma treated wounds at day 4 and 11 in Animal #2.

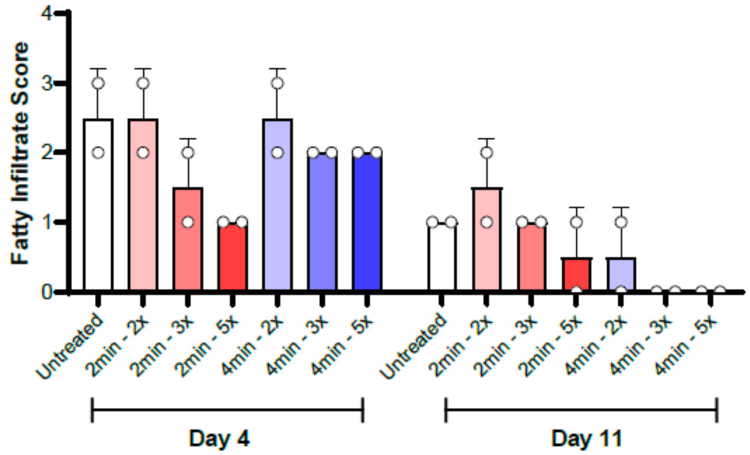

Supplementary Figure S15: Fatty infiltration scores between controls and plasma treated wounds at days 4 and 11 in Animal #2.

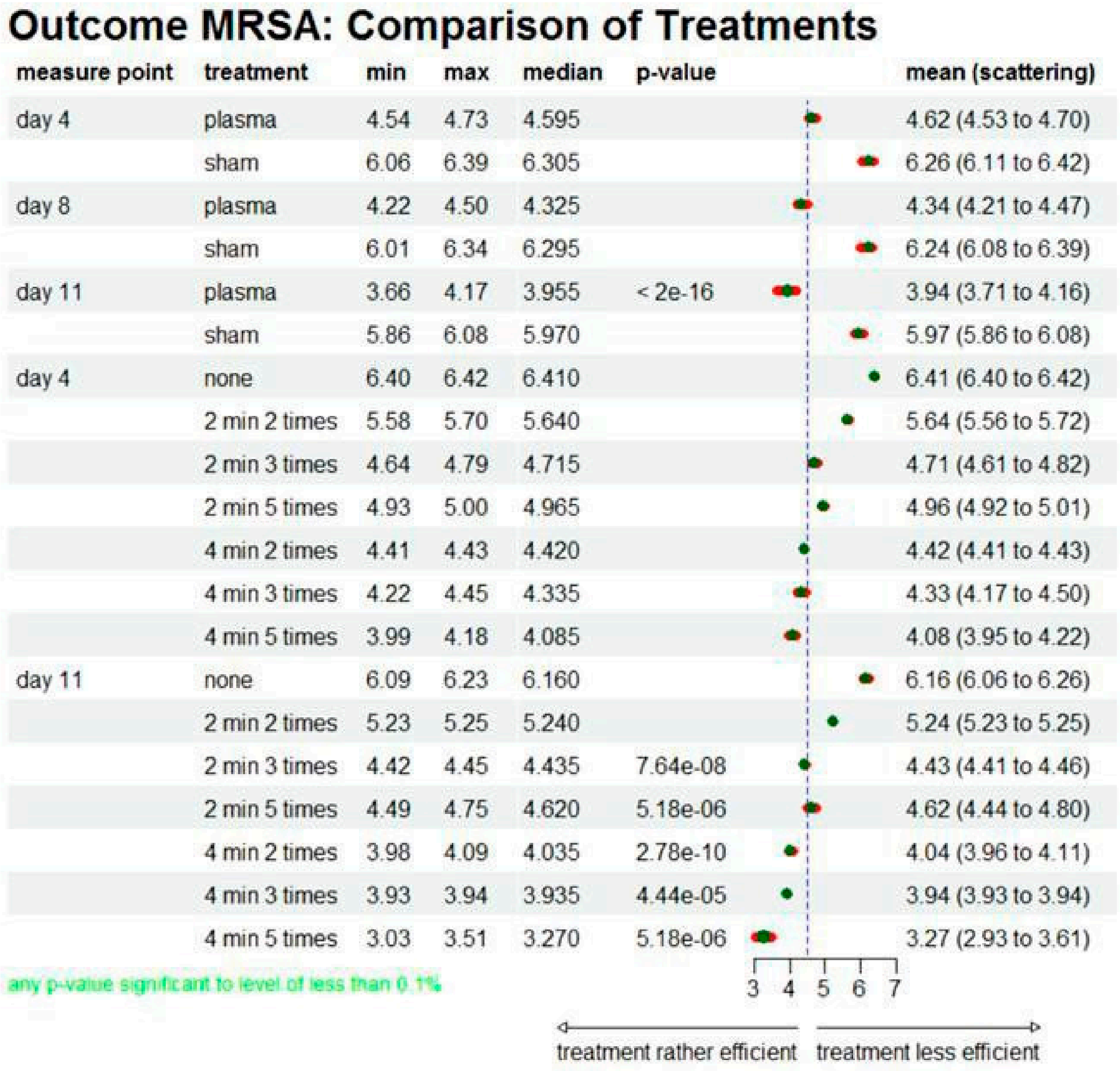

Outcome MRSA: Comparison of Treatments

measure point	treatment	min	max	median	p-value	mean (scattering)
day 4	plasma	4.54	4.73	4.595		4.62 (4.53 to 4.70)
	sham	6.06	6.39	6.305		6.26 (6.11 to 6.42)
day 8	plasma	4.22	4.50	4.325		4.34 (4.21 to 4.47)
	sham	6.01	6.34	6.295		6.24 (6.08 to 6.39)
day 11	plasma	3.66	4.17	3.955	< 2e-16	3.94 (3.71 to 4.16)
	sham	5.86	6.08	5.970		5.97 (5.86 to 6.08)
day 4	none	6.40	6.42	6.410		6.41 (6.40 to 6.42)
	2 min 2 times	5.58	5.70	5.640		5.64 (5.56 to 5.72)
	2 min 3 times	4.64	4.79	4.715		4.71 (4.61 to 4.82)
	2 min 5 times	4.93	5.00	4.965		4.96 (4.92 to 5.01)
	4 min 2 times	4.41	4.43	4.420		4.42 (4.41 to 4.43)
	4 min 3 times	4.22	4.45	4.335		4.33 (4.17 to 4.50)
	4 min 5 times	3.99	4.18	4.085		4.08 (3.95 to 4.22)
day 11	none	6.09	6.23	6.160		6.16 (6.06 to 6.26)
	2 min 2 times	5.23	5.25	5.240		5.24 (5.23 to 5.25)
	2 min 3 times	4.42	4.45	4.435	7.64e-08	4.43 (4.41 to 4.46)
	2 min 5 times	4.49	4.75	4.620	5.18e-06	4.62 (4.44 to 4.80)
	4 min 2 times	3.98	4.09	4.035	2.78e-10	4.04 (3.96 to 4.11)
	4 min 3 times	3.93	3.94	3.935	4.44e-05	3.94 (3.93 to 3.94)
	4 min 5 times	3.03	3.51	3.270	5.18e-06	3.27 (2.93 to 3.61)

any p-value significant to level of less than 0.1%

Supplementary Figure S16: MRSA Reduction Across Treatment Regimens following Cold Atmospheric Plasma (CAP) treatment.

Supplementary Table S1: Semi-quantitative Inflammatory Cell Infiltrate Scoring System					
	**Score**				
**Cell Type**	0	1	2	3	4
PMNs	0	1-5	6-10	Heavy	Packed
Lymphocytes	0	1-5	6-10	Heavy	Packed
Macrophages	0	1-5	6-10	Heavy	Packed
Giant Cells	0	1-2	3-5	Heavy	Packed
Mast Cells*	0	1-2	3-5	Heavy	Packed
* Toluidine Blue: mast cells & basophils					
**Tissue Responses**	0	1	2	3	4
Necrosis	0	Minimal	Mild	Moderate	Severe
Fibrosis	0	Narrow band	Moderate	Thick band	Extensive
Fatty Infiltrate	0	Minimal	Several layers	Broad	Extensive

Supplementary Table S2. Fluorescence and Histological Scoring Criteria

Endpoint	Category / Definition	Scale / Measurement
**Fluorescence Grade**	Grade 0: No red or cyan fluorescence signal detected<br>Grade 1: Localized or low-intensity red fluorescence within wound or periwound area, confined to anatomical borders<br>Grade 2: Strong or widespread red fluorescence extending beyond wound borders	Ordinal (0–2)
**Fluorescence (Binary)**	Negative: Grade 0<br>Positive: Grades 1–2	Binary (0 = negative; 1 = positive)
**Percent Re-epithelialization**	Proportion of wound surface covered with epithelium	0 = 0%<br>0.5 = 1–10%<br>1 = 11–30%<br>2 = 31–50%<br>3 = 51–70%<br>4 = 71–90%

Endpoint	Category / Definition	Scale / Measurement
		5 = 91–100%
**Epithelial Thickness**	Mean epithelial thickness measured at five equidistant points across the wound	Continuous (μm; mean of 5 measurements)
**White Cell Infiltration (WCI)**	Degree of inflammatory cell infiltration	1 = Absent<br>2 = Mild<br>3 = Moderate<br>4 = Marked<br>5 = Exuberant
**Granulation Tissue Formation**	Extent of granulation tissue formation within wound bed	Ordinal (0–5)

**Tissue Necrosis**	Presence and extent of necrotic tissue	0 = Absent; 5 = Extensive
**Fibrosis**	Degree of collagen deposition and extracellular matrix remodeling	0 = Absent; 5 = Extensive
**Fatty Infiltration**	Presence of adipocyte infiltration within tissue	0 = Absent; 5 = Extensive

Supplementary Table S3. Baseline patient characteristics

Characteristic	Patients (n=14)
Patients, n	14
Wounds, n	20
Age, median (IQR), years	79.5 (67.0 to 88.2)
Age, mean ± SD, years	76.9 ± 12.9
Female sex	5 (35.7%)
Male sex	9 (64.3%)

Characteristic	Patients (n=14)
Patients with 1 wound	9 (64.3%)
Patients with 2 wounds	4 (28.6%)
Patients with 3 wounds	1 (7.1%)
Diabetes mellitus: yes	6 (42.9%)
Hypertension: yes	10 (71.4%)
Hypertension: unknown	1 (7.1%)
Anemia: yes	3 (21.4%)
Cardiovascular disease: yes	8 (57.1%)
Chronic kidney disease: yes	4 (28.6%)
Chronic lung disease: yes	4 (28.6%)
Vascular disease: yes	3 (21.4%)
Deep vein thrombosis: yes	2 (14.3%)
Smoking status: never	6 (42.9%)
Smoking status: former	4 (28.6%)
Smoking status: current	2 (14.3%)
Smoking status: unknown	2 (14.3%)
Alcohol use: no	6 (42.9%)
Alcohol use: yes	5 (35.7%)
Alcohol use: unknown	3 (21.4%)

Percentages use the patient denominator $n = 14$.

Supplementary Table S4. Baseline wound characteristics

Characteristic	Wounds (n=20)
Wounds, n	20
**Characteristic**	**Wounds (n=20)**
Venous ulcers	15 (75.0%)
Mixed lymphovenous ulcers	2 (10.0%)
Arterial ulcers	1 (5.0%)
Lymphatic ulcers	1 (5.0%)
Traumatic ulcers	1 (5.0%)
Area at study entry (week 1), median (IQR), cm^2	12.1 (5.8 to 16.7)
Area at study entry (week 1), mean ± SD, cm^2	12.16 ± 7.94
Analyzable MolecuLight fluorescence series	17 (85.0%)
Excluded MolecuLight fluorescence series	3 (15.0%)
Baseline fluorescence at ML1 among analyzable series: High (grade 2)	13 (76.5%)
Baseline fluorescence at ML1 among analyzable series: Moderate (grade 1)	4 (23.5%)
Baseline fluorescence at ML1 among analyzable series: None (grade 0)	0 (0.0%)

Percentages use the wound denominator n = 20. Baseline fluorescence distribution is shown only for the 17 analyzable series.

Supplementary Table S5. De-identified wound-level baseline data and MolecuLight dataset completeness

De-ID wound	De-ID patient	Study wound code	Ulcer type	Study-entry area, week 1 (cm^2)	Concomitant dressing category during CAP phase	Baseline ML1 grade	Included in fluorescence analysis	Reason if excluded
W01	P01	401	Lymphatic	6.40	Silver-based	2	Yes	
W02	P02	402	Venous	4.20	Neutral	2	Yes	
W03	P03	403	Trauma (insect bite)	11.30	Neutral		No	Images were recorded at all five visits, but no finalized ordinal grades were retained in the final grading workbook.
W04	P04	404	Mixed - Lymphovenous	3.90	Silver-based	1	Yes	
W05	P04	405	Mixed - Lymphovenous	4.10	Silver-based	1	Yes	
W06	P05	406	Arterial	16.60	Iodine-based	2	Yes	

De-ID wound	De-ID patient	Study wound code	Ulcer type	Study-entry area, week 1 (cm²)	Concomitant dressing category during CAP phase	Baseline ML1 grade	Included in fluorescence analysis	Reason if excluded
W07	P06	407	Venous	16.80	Neutral	2	Yes	
W08	P07	408	Venous	5.40	Neutral	2	Yes	
W09	P07	409	Venous	19.80	Neutral	2	Yes	
W10	P08	410	Venous	32.00	Silver-based	1	Yes	
W11	P09	411	Venous	25.00	Silver-based; Hydrogel	2	Yes	
W12	P10	412	Venous	12.90	Silver-based	2	Yes	
W13	P11	413	Venous	15.60	Silver-based	1	Yes	
W14	P12	414	Venous	9.70	Silver-based; Steroid adjunct	2	Yes	
W15	P12	415	Venous	13.70	Silver-based; Neutral; Steroid adjunct	2	Yes	
W16	P13	416	Venous	5.90	Not coded; Steroid adjunct		No	Incomplete 5-visit series
W17	P13	417	Venous	18.90	Iodine-based; Steroid adjunct	2	Yes	
W18	P13	418	Venous	6.10	Iodine-based; Steroid adjunct		No	Images were recorded at all five visits, but no finalized ordinal grades were retained in the final grading workbook.
W19	P14	419	Venous	1.20	Silver-based; Steroid adjunct	2	Yes	
W20	P14	420	Venous	13.70	Silver-based; Steroid adjunct	2	Yes	

This table documents cohort linkage and fluorescence dataset completeness; wound-area reporting is limited to study-entry baseline.

Supplementary Table S6. Concomitant standard wound care protocol

Domain	Control phase (weeks 1-4)	CAP phase (weeks 5-9)	Follow-up phase (weeks 10-13)
Visit schedule	Weekly assessment during 4-week control phase.	Twice-weekly CAP visits for 5 weeks (10 treatments total); standard care delivered with each visit.	Standard care continued during follow-up; no CAP applied.

Domain	Control phase (weeks 1-4)	CAP phase (weeks 5-9)	Follow-up phase (weeks 10-13)
Cleansing	Cleansing with polyhexanide (PHMB) plus betaine surfactant (Prontosan®), octenidine/allantoin wipes (Octenilin®), and povidone-iodine solution (Videne®).	Same cleansing approach as control phase, performed before CAP treatment.	Same as control phase.
Debridement	Superficial debridement when clinically indicated.	Debridement when necessary before CAP application.	Debridement when clinically indicated.
Dressings	Non-occlusive dressings selected according to local protocol and wound condition.	Appropriate dressing selected after CAP treatment according to clinical indication.	Dressing application continued according to local protocol.
Offloading / supportive care	Standard supportive wound care according to clinical judgment.	Offloading added where clinically indicated; no protocol-mandated new healing adjuncts introduced.	Standard supportive wound care according to clinical judgment.
Concurrent topical antibiotics	No concurrent topical antibiotics were introduced during the intervention phase.		
Assessments linked to SWC	Digital photography and wound-area measurement weekly; control-phase fluorescence imaging was planned in the thesis text but no analyzable ordinal dataset was retained outside the intervention phase.	Digital photography, wound-area measurement, MolecuLight fluorescence imaging, and pain scoring performed at intervention visits.	Digital photography and wound-area measurement continued; no CAP administered.

Protocol elements were reconstructed from the thesis methods text. Per-visit brand-level dressing logs were not retained beyond the categorical exposure coding summarized in Table S7.

Supplementary Table S7. Observed dressing and supportive care exposure during the CAP phase

Observed concomitant care component	Wounds, n	% of 20 wounds
Silver-based dressing used at any point	11	55.0%
Iodine-based dressing used at any point	3	15.0%

Observed concomitant care component	Wounds, n	% of 20 wounds
Neutral/non-antimicrobial dressing used at any point	6	30.0%
Hydrogel used at any point	1	5.0%
Topical steroid adjunct used at any point	7	35.0%

Counts are non-mutually exclusive because some wounds received combination dressings or a steroid adjunct.

Supplementary Table S8. Raw analyzable MolecuLight fluorescence grades

De-ID wound	De-ID patient	Study wound code	Ulcer type	Area at ML1 (cm²)	ML1	ML2	ML3	ML4	ML5	Concomitant dressing category
W01	P01	401	Lymphatic	7.60	2	1	1	1	1	Silver-based
W02	P02	402	Venous	3.40	2	2	2	2	0	Neutral
W04	P04	404	Mixed - Lymphovenous	5.70	1	1	1	0	0	Silver-based
W05	P04	405	Mixed - Lymphovenous	0.70	1	0	0	0	0	Silver-based
W06	P05	406	Arterial	39.10	2	2	2	2	1	Iodine-based
W07	P06	407	Venous	6.50	2	2	2	1	0	Neutral
W08	P07	408	Venous	1.70	2	0	2	1	0	Neutral
W09	P07	409	Venous	2.50	2	0	1	2	0	Neutral
W10	P08	410	Venous	24.90	1	1	1	1	1	Silver-based
W11	P09	411	Venous	20.30	2	2	2	1	0	Silver-based; Hydrogel
W12	P10	412	Venous	14.50	2	2	1	1	2	Silver-based
W13	P11	413	Venous	12.10	1	1	1	2	0	Silver-based
W14	P12	414	Venous	9.00	2	2	1	2	0	Silver-based; Steroid adjunct
W15	P12	415	Venous	10.30	2	2	2	2	0	Silver-based; Neutral; Steroid adjunct
W17	P13	417	Venous	28.30	2	2	2	2	1	Iodine-based; Steroid adjunct
W19	P14	419	Venous	1.40	2	2	2	0	0	Silver-based; Steroid adjunct

De-ID wound	De-ID patient	Study wound code	Ulcer type	Area at ML1 (cm²)	ML1	ML2	ML3	ML4	ML5	Concomitant dressing category
W20	P14	420	Venous	16.90	2	2	2	0	0	Silver-based; Steroid adjunct

Grades are the finalized ordinal fluorescence scores retained in the MolecuLight grading workbook: 2 = high, 1 = moderate, 0 = none.

Supplementary Table S9. MolecuLight series excluded from repeated-measures analysis

De-ID wound	De-ID patient	Study wound code	Ulcer type	Weekly image log (weeks 5-9)	Reason for exclusion
W03	P03	403	Trauma (insect bite)	Y ǀ Y ǀ Y ǀ Y ǀ Y	Images were recorded at all five visits, but no finalized ordinal grades were retained in the final grading workbook.
W16	P13	416	Venous	Y ǀ N ǀ Y ǀ Y ǀ Y	Incomplete 5-visit series
W18	P13	418	Venous	Y ǀ Y ǀ Y ǀ Y ǀ Y	Images were recorded at all five visits, but no finalized ordinal grades were retained in the final grading workbook.

Y indicates image acquisition recorded in the weekly log; N indicates no gradable/available fluorescence image recorded. For W03 and W18, image acquisition was logged across five visits, but no finalized ordinal grades were retained in the final grading workbook.
